# Likelihood ratios: Clinical application in day-to-day practice

**DOI:** 10.4103/0301-4738.49397

**Published:** 2009

**Authors:** Rajul Parikh, Shefali Parikh, Ellen Arun, Ravi Thomas

**Affiliations:** 1 Bombay City Institute and Research Center, Mumbai, India; 2 Sadhuram Eye Hospital, Hyderabad, India; 3 Prime Medical Center, Dubai, UAE; 4 Queensland Eye Institute; 5 University of Queensland; 6 Visiting Professor, L. V. Prasad Eye Institute, Hyderabad, India

**Keywords:** Likelihood ratio, pretest probability, pretest odds, posttest odds, posttest probability

## Abstract

In this article we provide an introduction to the use of likelihood ratios in clinical ophthalmology. Likelihood ratios permit the best use of clinical test results to establish diagnoses for the individual patient. Examples and step-by-step calculations demonstrate the estimation of pretest probability, pretest odds, and calculation of posttest odds and posttest probability using likelihood ratios. The benefits and limitations of this approach are discussed.

In an earlier article, we explained how we use sensitivity, specificity, positive and negative predictive values in day-to-day practice.[1] While the clinical application of sensitivity and specificity is useful, they have limitations. With sensitivity and specificity, we use a cutoff point to divide the test into (only) two results: positive or negative. In real life, diseases present in gradations of severity; by limiting a test result to “positive” or “negative”, we stand to lose important diagnostic information. Sensitivity and specificity are independent of disease prevalence, but are dependent on disease severity.[2,3] In early disease, it is difficult to differentiate between health and illness and the sensitivity decreases; it increases in severe disease. Accordingly, the reported sensitivity and specificity for a disease may not always reflect the sensitivity and specificity for the individual patient. Additionally, disease prevalence has a significant impact on the positive predictive value (PPV) and negative predictive values (NPV).[4] Therefore, despite a high sensitivity and specificity of a given test, the PPV will be very low in a disease with a very low prevalence.[1]

As a “bad” example of clinical practice, suppose a colleague measures an intraocular pressure (IOP) of 22 mm Hg (recorded once) on a 45-year-old male and orders a nerve fiber layer imaging with glaucoma diagnostics variable corneal compensator (GDx VCC, scanning laser polarimetry), or any other imaging modality. (We chose the GDx VCC as its parameter “NFI (nerve fiber index)” is easy to use in the examples that follow). We consider this a “bad” example of clinical practice, because such tests obtained after a comprehensive examination (and confirmation of the IOP values) have far more significance. Nevertheless, the machine provides a nice printout and a value for the “NFI” of say 48. The literature indicates that an NFI of 50 is highly suggestive for glaucoma[5] but does that establish our diagnosis? The NFI value varies from 0 – 100. Dividing this continuous scale into two zones at an arbitrary cutoff value of 30 (or 50 as is suggested for “definite” pathology) has limitations.

(Multilevel) likelihood ratios (LRs) overcome the disadvantage of a single cutoff and allow us to best apply the results of diagnostic tests to the individual patient.[4] In this article we will explain LRs as well and use clinical examples to illustrate their use. In order to understand LR, we need to revise the concepts of sensitivity and specificity discussed in an earlier article.[1]. We will briefly summarize these terms here and move on.

In the conventional 2×2 Table shown in Table 1 Sensitivity = a / a+c

**Table 1:** Sensitivity and specificity

	Gold standard (Disease present)	Gold standard (Disease absent)		
Test	True	False	Total	PPV:
positive	positives (TP) a	positives (FP) b	Test positives: a+ b	a / (a+b)
Test	False	True	Total	NPV:
negative	negative (FN)	negatives (TN)	Test negatives:	d/ (c+d)
	c	d	c+d	
	Total	Total	Total Population:	
	Diseased:	Normal:	a+b+c+d	
	a+c	b +d		
	Sensitivity:	Specificity:		
	a/ (a+c)	d/ (b+d)		

PPV: Positive predictive value, NPV: Negative predictive value

= a (true positive) / a+c (true positive + false negative) Specificity = d / b+d

= d (true negative) / b+d (true negative + false positive) Positive Predictive Value (PPV): = a / a+b

= a (true positive) / a+b (true positive + false positive)

= Probability that the patient has disease when test is positive.

NPV Negative Predictive Value (NPV): = d / c +d

= d (true negatives) / c+d (false negative + true negative)

= Probability that the patient does not have disease when test is negative.

Prevalence (in a clinical situation this is referred to as pretest probability) of a disease is the proportion of patients with the target disorder in the population tested.

= a+c / (a+b+c+d)

Notice in Table 1 that sensitivity and specificity are calculated “vertically” while the PPVs are calculated horizontally. Accordingly, PPVs are influenced by the number of patients in the columns; we can alter this by changing the number of diseased and controls. In other words, the PPV is affected by prevalence (pretest probability) of the disease; sensitivity and specificity are not.

## Definition of LR

The LR is the probability of a given test result in a patient with the target disorder divided by the probability of that same result in a person without the target disorder. The components of the LR are calculated vertically, and like the sensitivity and specificity are immune to the prevalence.

The LR of a positive test result:

LR+ = probability that an individual with the target disorder has a positive test probability than an individual without the target disorder has a positive test

In other words, LR+ = True positivity rate / False positivity rate

Which is the same as sensitivity / 1- specificity

It is appropriate at this stage to introduce and explain certain terms we need to use LR:

Pretest Probability is defined as the probability of the target disorder before a diagnostic test is ordered. In the “general” population, it would be called the prevalence of the disease. For example, the prevalence of primary open angle glaucoma (POAG) in the south Indian population above 40 years of age is approximately 2.5%.[6] Accordingly, any patient in this age group who walks into a general ophthalmology clinic has a 2.5% probability of glaucoma even before the history or examination. In the clinic, the “prevalence” of a disease before ordering a test is called the pretest probability. If this is done without taking a history or examining the patient, the pretest probability is about the same as the prevalence of the disease in the population.

Pretest odds: The odds that the patient has the target disorder, before the test is carried out. It is slightly different from and is calculated from pretest probability.

Pretest odds: (pretest probability/ [1 – pretest probability]).

Posttest odds: (pretest odds × LR): The odds that the patient has the target disorder, after the test results are known. It is calculated by multiplying the pretest odds by the likelihood of a positive or negative test (as we will show).

Posttest probability is defined, as the probability of the target disorder after a diagnostic test result is known. It is slightly different from and calculated from posttest odds.

Post-test probability: (posttest odds/[Posttest odds + 1]).

The advantage of the LR is that we can multiply the pretest odds that the patient has disease by the LR of a positive test to obtain the posttest odds that the patient has disease. This of course means that we must first estimate the pretest probability (and pretest odds) of disease. As mentioned, prior to the history or examination, the pretest probability of disease is the same as the prevalence of the disease in the general population. Once the history and examination are completed, the pretest probability may remain the same, decrease, or is revised upwards. This seems complicated, but clinicians do this intuitively all the time; with a little experience they can learn to quantify their gut feeling. The pretest probability is converted to the pretest odds. The pretest odds are multiplied by the LR to provide the posttest odds.

Posttest odds = pretest odds * LR

Finally the posttest odds are converted to the posttest probability of disease.

## Clinical application

Let us come back to our example of “bad” clinical practice: Our colleague has recorded an intraocular pressure (IOP) of 21 mm Hg on a 45-year-old patient and ordered a GDx VCC. The machine provides a nerve fiber indicator (NFI) score of 48. Does this patient have POAG?

The information that we have is

1. The pretest probability of POAG. Without any other information, this is the same as the prevalence of POAG in 45-year-olds in the given population. We will use published literature from South India for this purpose: 2.5%.[6]
2. Clinical information:
  IOP: 22 mm Hg in both eyes
3. Sensitivity and specificity of GDx:
  GDx VCC (NFI score 48): For our patient at this cutoff using our published data, sensitivity is 59.5% and specificity 97.1%.[7] (For calculation purposes we'll use 60% sensitivity and 97% specificity.)

We can now calculate the LR for the test result:

GDx VCC (for NFI score 48)

LR +ve = Sensitivity / 1- specificity = 0.6 / 0.03 = 20

We now have the positive LR ratio as well as the pretest probability (in this case, the population prevalence) of POAG. As the IOP is at the upper limit of normal as per Indian population-based data,[8] has not been rechecked, and we do not know the corneal thickness, we will not take it into account, and go to the next step.

The pretest odds = pretest probability / (1-pretest probability)

As the pretest probability is the same as the prevalence of POAG in the population,

the pretest odds: 0.025 / 0.975 = 0.03.

Posttest odds = pretest odds * LR

= 0.03 * 20 = 0.6

We now calculate posttest probability.

Posttest probability = posttest odds / (posttest odds+1)

Therefore the Posttest probability = 0.6/ 1.6 = 0.375 or 37.5%.

The result means that after the GDx VCC results, the probability of our patient having glaucoma has increased from 2.5% to 38%. A probability of 38% is worse than obtaining a heads or tails on the random toss of a coin; certainly not good enough to make a diagnosis of glaucoma. This example also demonstrates that one test in isolation, even a good one like the GDx, even if strongly positive, may not confirm the diagnosis. A comprehensive eye examination with judicial use of the GDx (or any other imaging technology) is more helpful.

Let's try the same example with a slight difference: The IOP is now “high” (24 mm Hg, confirmed on several readings; “corrected” for corneal thickness). GDx VCC printout shows an NFI score of 48 as above.

Sensitivity and specificity of IOP: (50% sensitivity and 92% specificity[9])

Positive LR of IOP: = sensitivity / 1- specificity = 0.5/ 100 − 92 = 0.5 /.08 = 6.25

LR NFI at cutoff 48: 20

From theabove example, we know that the pretest probability of POAG before examination was 2.5% and the pretest odds 0.03. How much does this change with a raised IOP? For that we need to calculate posttest probability of POAG using the LR ratio of a raised IOP.

Posttest odds = pretest odds * LR for IOP

So, posttest odds = 0.03 * 6.25 = 0.188

We now calculate posttest probability:

Posttest probability = posttest odds / (posttest odds+1)

So, posttest probability = 0.188 / 1.188 = 0.16

That means that after our “high” IOP measurement, the probability of our patient having POAG has increased from 2.5% to 16%. Not good enough to make a diagnosis of glaucoma.

We now add on the GDx VCC results.

Pretest probability before GDX= 16%

Pretest odds: 0.16 / 0.84 = 0.19

LR of GDx result of NFI 48= 20

Posttest odds = 0.19 * 20 = 3.8

Posttest probability = 3.8 / 4.8 = 0.792

After IOP measurement and GDx VCC results data, the probability that our patient has glaucoma has increased from 2.5% to 79%. Still not good enough to clinch the diagnosis.

In the third example, we order the test after a full clinical examination; the way it should be done. The findings are an IOP of 24 mm Hg in both eyes, open angles, other pathology has been ruled out and the disc has been examined stereoscopically using biomicroscopy. The cup disc ratio was 0.7:1 disc ratio in a medium-sized disc, with inferior rim thinning with a wedge-shaped inferior retinal nerve fiber layer (RNFL) defect. The GDx VCC NFI score is 48. How do we use this information for the patient?

### First we calculate LR for each test:

1. IOP: as above = 6.25
2. Optic disc: sensitivity (20%), specificity (99%).[10] Using the same formula: LR + = sensitivity / 1- specificity = 0.2 / 0.01 = 20
3. GDx VCC (for NFI score > 48): LR + = 20.

After IOP measurement, the probability that our patient has POAG had increased to 16%. This 0.16 becomes the pretest probability for our second calculation that is optic disc examination. We now incorporate optic disc findings into the calculation.

Pretest odds: pre test probability / 1 minus pretest probability = 0.16 / 0.84 = 0.188

Posttest odds = pretest odds × LR = 0.188 * 20 = 3.76

Posttest probability = posttest odds / posttest odds +1 = 3.76 / 4.76 = 0.79

After IOP measurement and optic disc assessment, the probability that our patient has POAG has increased from 2.5% through 16% (after the raised IOP), to 79% after incorporating optic disc finding also. To make a diagnosis so as to start treatment, or at least to tell the patient, we may want to be 90 plus % sure. (How sure we need to be of a diagnosis varies with the disease, the examiner and the patients and is beyond the scope of this article. The reader is referred to our clinical bible[4].) Be that as it may, we now add the GDx VCC findings.

This 0.79 becomes the pretest probability for our third calculation that is GDx VCC.

Pretest odds: 0.79 / 0.21 = 3.76

Now add LR of NFI: 20

Posttest odds = 3.76 * 20 = 75.2

Posttest probability = 75.2 / 76.2 = 0.987

In other words, after incorporating the GDx result, we are now 98.7% sure that our patient has POAG.

We have calculated the pretest odds and posttest probability for each stage of the case step by step. This was done in order to familiarize ourselves with these calculations. Fortunately, in practice, we do not have to follow this lengthy route. Once, we have LRs for the various signs, symptoms and tests, we can directly calculate the final posttest odds using the following formula.

Posttest Odds = Pretest Odds × LR_1_ × LR_2_ × LR_3_ … × LR_n_.

For the above example:

Posttest Odds = 0.03 × 6.25 (LR for IOP) × 20 (LR for Disc) × 20 (LR for GDX) = 75.

And, posttest probability = Posttest odds / Posttest odds +1 = 75/ 76 = 98.7%

## Negative likelihood ratio

The LR of a negative test result (LR-) is described in most texts as

LR- = probability that an individual with the condition has a negative test /probability than an individual without the condition has a negative test

LR - = 1- sensitivity / specificity

We prefer to determine the probability of being normal and use the following formula:

LR - = Specificity / 1- sensitivity

This to us is more intuitive and symmetrical to the formula for positive LR.

### Example

A colleague examines a 54-year-old patient with IOP of 20 mm Hg (recorded twice) and orders a GDx VCC, scanning laser polarimetry. The machine provides an NFI score of 18. What are the chances that this patient is normal? We can now easily perform the calculations for that situation, but as discussed above, using the imaging test alone is not good clinical practice. And as space is limited, we will use an example with the clinical information available after a comprehensive eye examination.

### What is the information we need?

1. Prevalence of ocular hypertension (OHT) and glaucoma (POAG and primary angle closure glaucoma (PACG)) in a given population. The published literature from south India provides the information.
  Prevalence of POAG + OHT + POAG suspect + PACG: 5% (APEDS data)[6,11]
2. Clinical information:
  Family history of glaucoma: Nil
  Gonioscopy: open angles
  IOP: 20 mm Hg in both eyes
  Optic disc: normal disc size, 0.4 cup to disc ratio, healthy neuroretinal rim (following ISNT rule)
3. Sensitivity and specificity of each test performed
  IOP: At 21 mm Hg cutoff: Sensitivity: 50%, Specificity: 92% (Baltimore Survey data)[9]
  Optic disc (for ISNT rule): 72% sensitivity, 79% specificity10 (reference number should be 10
  GDx VCC score of 20: Sensitivity: 90.5%, Specificity 52.9%[7]

### We can calculate LR for each test:

1. IOP: LR - = Specificity / 1- sensitivity.
  = 0.92 / 1 - 0.5 = 0.92/ 0.5 = 1.84
2. Optic disc: Using same formula: LR - = 0.79 / 0.28 = 2.82
3. GDx VCC (for NFI score > 20): LR - = 0.53 / 0.095 = 5.6

How do we use this negative LR ratio? When the patient walked into the clinic the probability of having the disease was the same as a prevalence of a given disease in the population. Here the particular disease is “glaucoma suspect” (which includes ocular hypertension, glaucoma suspect and definite POAG and PACG) and prevalence is 5%. In other words, the chances that our patient is normal are 1 minus the probability of disease or 100 minus 5% = 95% = 0.95. Pretest odds are pretest probability / 1- pretest probability = 0.95/0.05 = 19. After initial examination and with a normal IOP, what are the chances that the patient is normal? We need to calculate the posttest probability using pretest probability and LR ratio. The formulae are mentioned and explained above.

We will use Posttest Odds = Pretest Odds × LR_1_ × LR_2_ × LR_3_ … × LR_n_.

Posttest Odds = 19 × 1.84 × 2.82 × 5.6 = 552

Posttest probability = 552 / 553 =.998 = 99.8%

Incidentally, if we had used just the clinical assessment (IOP and optic disc assessment), the probability of our patient being normal would have increased from 95% to 99%. The reader is welcome to calculate that, but that is about as clinically certain as we can get. Using GDx that surety increased to 99.8%. The value of an increase in certainty from 99.5 to 99.8% is debatable.

We don't have to remember all these formulae and definitely don't need to go through complicated calculations. We can also use a nomogram to calculate the posttest probability.[12] What we need to know is prevalence of disease and the sensitivity and specificity of the test for the value obtained for the individual patient. We only have to calculate LR.

Table 2 provides general guidelines about different cutoff values of LR ratio. In general LR values of 10 are considered significantly large.

**Table 2:** Interpretation of LR ratio for various values

LR	Interpretation
> 10	Large and often conclusive increase in the likelihood of disease
5–10	Moderate increase in the likelihood of disease
2–5	Small increase in the likelihood of disease
1–2	Minimal increase in the likelihood of disease
1	No change in the likelihood of disease

## Advantages of the likelihood ratio approach

- LRs can deal with tests with more than two possible results (not just normal/abnormal). LRs using multiple “levels” provide useful information about the individual patient's test result. To calculate the LR, we must know the sensitivity and specificity of a test at various cutoff levels. Ideally, multilevel LRs for various cutoff levels for their test results should be provided by the manufacturers; we can also obtain / calculate this information from the literature.
- The magnitude of the LR provides an intuitive feeling for how strongly a given test result will raise (rule-in) or lower (rule-out) the likelihood of disease. The LR+ corresponds to the clinical concept of “ruling-OUT disease”. If the LR of a test is very high and such a test is negative, it rules out disease. The LR- corresponds to the clinical concept of “ruling-IN disease”. If the LR of a negative test is very low, and such a test is positive, it rules in disease.
- The LR+ and LR- do not change as the underlying probability of disease changes (unlike predictive values.)
- Computing posttest odds after a series of diagnostic tests is much easier than using the sensitivity/specificity method. Posttest Odds = Pretest Odds × LR_1_ × LR_2_ × LR_3_… × LR_n_.

LRs are probably the best way to utilize diagnostic data, but do have limitations. One limitation is related to estimation of pretest probabilities. Another is the wide confidence intervals around the LRs, especially the ones that are capable of ruling in or ruling out a diagnosis. This is due to paucity of data at the extremes of the disease spectrum where the LRs are likely to be the most helpful. Finally, as the LRs are calculated from the sensitivity and specificity, like these parameters they too may be affected by severity of disease.

One way to work around some of these limitations is to perform a “sensitivity analysis” using different, sensible pretest probabilities. These pretest probabilities can be what members of the clinical team consider to be reasonable after a clinical exam. If the lowest “sensible” pretest probability still provides a posttest probability of 90% or more we can be “sure” of our diagnosis. A similar process can be used to rule out the diagnosis.

## Summary

Based on the patient's history and clinical examination we estimate the pretest probability of disease and calculate the pretest odds from that. We then multiply the pretest odds by the LR of the test result for that individual patient (multilevel LR) to obtain the posttest odds. Finally, we convert the posttest odds to the more clinically intuitive posttest probability. Using this information we can get as close to a “rule in” or “rule out” criteria for our individual patient.

This approach of using LR to calculate the posttest probability of disease makes best use of diagnostic information. It is however not required for every case and certainly not for straightforward cases. For example, a patient with IOP of 32 mm Hg and 0.9 cup to disc ratio in a medium-sized disc with a bipolar notch does not need any calculation to make the diagnosis. But the intuitive process underlying this obvious diagnosis is applicable via LRs to difficult cases where there is diagnostic dilemma. A patient with suspected pre-perimetric glaucoma, for example, is a good case to use LR. It is also important to remember that there is a limit to testing: we can never be absolutely certain. As our clinical Bible states: “physicians must be content to end not in certainties, bur rather in statistical probabilities. The modern physician has right to be certain, within statistical constraints, but never cocksure. Absolute certainties remain for some theologians – and likeminded physicians.”[4,13]

## References

[1] Parikh RS, Mathai A, Parikh S, Chandra Sekhar G, Thomas R (2007). Understanding and using sensitivity, specificity and predictive values. Indian J Ophthalmol.

[2] Moons KG, van Es GA, Deckers JW, Habbema JD, Grobbee DE (1997). Limitations of sensitivity, specificity, likelihood ratio, and Bayes' theorem in assessing diagnostic probabilities: A clinical example. Epidemiology.

[3] Brenner H, Gefeller O (1997). Variation of sensitivity, specificity, likelihood ratios, and predictive values with disease prevalence. Stat Med.

[4] Sackett DL, Haynes RB, Guyatt GH, Tugwell P (1991). Clinical Epidemiology. A Basic Science for Clinical Medicine.

[5] GDx Primer, Chapter 3. The Normative Database, page 27.

[6] Dandona L, Dandona R, Srinivas M, Mandal P, John RK, McCarty CA (2000). Open-angle glaucoma in an urban population in southern India the Andhra Pradesh Eye Disease Study. Ophthalmology.

[7] Parikh RS, Parikh SR, Prabakaran S, Ganesh Babu J, Thomas R (2008). Diagnostic Capability of scanning laser polarimetry (GDx VCC) in Early Glaucoma. Ophthalmology.

[8] Vijaya L, George R, Baskaran M, Arvind H, Raju P, Ramesh SV (2008). Prevalence of Primary Open-angle Glaucoma in an Urban South Indian Population and Comparison with a Rural Population The Chennai Glaucoma Study. Ophthalmology.

[9] Teilsch JM, Katz J, Singh K, Quigley HA, Gottsch JD, Javitt J (1991). A population based evaluation of glaucoma screening: The Baltimore Eye Survey. Am J Epidemiol.

[10] Jonas JB, Schiro D (1994). Localised wedge shaped defects of the retinal nerve fibre layer in glaucoma. Br J Ophthalmol.

[11] Dandona L, Dandona R, Mandal P, Srinivas M, John RK, McCarty CA (2000). Angle-closure glaucoma in an urban population in southern India: the Andhra Pradesh Eye Disease Study. Ophthalmology.

[12] Fagan TJ (1975). Nomogram for Bayes's theorem. N Engl J Med.

[13] Spodick DH (1975). On expert and expertise: The effect of variability in observer performance. Am J Cardiol.

